# Conformally Mapped Multifunctional Acoustic Metamaterial Lens for Spectral Sound Guiding and Talbot Effect

**DOI:** 10.34133/2019/1748537

**Published:** 2019-11-12

**Authors:** He Gao, Xinsheng Fang, Zhongming Gu, Tuo Liu, Shanjun Liang, Yong Li, Jie Zhu

**Affiliations:** ^1^Department of Mechanical Engineering, The Hong Kong Polytechnic University, Hung Hom, Kowloon, Hong Kong SAR, China; ^2^Institute of Acoustics, School of Physics Science and Engineering, Tongji University, Shanghai 200092, China; ^3^The Hong Kong Polytechnic University Shenzhen Research Institute, Shenzhen 518057, China

## Abstract

We demonstrate a conformally mapped multifunctional acoustic metamaterial Mikaelian lens. Mikaelian lens is a gradient medium with a hyperbolic secant refractive index profile that can realize functions like beam self-focusing. Unlike the conventional design approaches, with a conformal transformation method, only isotropic material parameters with gradient refractive index profiles are required for the construction of such lens. To realize desired gradient index distribution, we carefully design a new type of cross-channel-shaped acoustic metamaterial, whose refractive index can be effectively modulated by simply changing the slit opening size. The distinct capabilities of the metamaterial Mikaelian lens in manipulating acoustic waves are experimentally verified with the fabricated sample. Simultaneous sound guiding and Talbot effects, which normally require respective geometrical and wave acoustic approximations, are observed in simulations and experiments. Furthermore, those effects of shaping acoustic wave propagations were validated within a relatively wide frequency range. Our study reveals how the conformal transformation method can help to bridge the ray acoustics with wave acoustics. It offers opportunities to the development of novel multifunctional acoustic devices for various applications, such as sound and particle manipulations.

## 1. Introduction

Acoustic metamaterials exhibit novel constitutive properties that extensively broaden the acoustic research horizon. They offer the possibility to manipulate sound in unprecedented ways to significantly benefit potential applications [1–19]. A generalized way to appreciate the implied capabilities of acoustic metamaterials is through the concept of transformation acoustics, which enables freewheeling steering of acoustic rays along arbitrary curves [7–15]. This coordinate transformation method was first proposed to manipulate electromagnetic waves, which is based on the invariance of Maxwell's equations under coordinate transformations [20–23]. Similar invariance symmetry of the acoustic wave equations under transformation was then identified [8]. This landmark finding thus enabled the establishment of the general principle of transformation acoustics. Transformation acoustics offers a variety of versatile and interesting sound control functionalities, among which, cloaking is one of the most remarkable and fascinating phenomenon [7–9]. However, the unit parameters of sound cloaking acoustic metamaterials are usually extreme. In addition, the effective parameter values derived from transformation acoustics usually are highly dispersive, limiting the working frequency range. To overcome the limitations, simplified schemes were proposed. For example, based on the quasiconformal mapping method [10, 11], carpet cloaks were able to hide scatters on a flat plane. The conformal transformation method was also applied to design the isotropic media only spherical cloak [16].

The transformation function of quasiconformal mapping approximately satisfies the requirement of conformal mapping to reduce the anisotropic feature of material parameters. Such conformal transformation method shows great advantages when it comes to constructing practical devices, as it only requires an isotropic parameter with gradient refractive index profiles. It therefore can bring the benefit of feasible material design, which is not easy to be realized with transformation acoustics, to applications such as an invisibility device [24], whispering gallery modes [25], and perfect lensing [26]. Moreover, the conformal transformation method does not require the use of resonant metamaterials, which implies that the resulting metamaterial device can have a much improved working bandwidth.

According to Fermat's principle, a spatially varied refractive index leads to bended travelling rays. The propagating path of the acoustic wave can be tuned in a desired way by changing the refractive index with space. This is the fundamental consensus of geometrical approaches. However, with the geometric acoustic approach, acoustic waves are considered as rays, what is important is the propagating trajectories. While from the aspect of wave acoustics, both trajectories and phase changes matter. Previously, materials and devices with different functionalities were designed based on either the geometric [27–29] or the wave acoustics [30–32]. Therefore, any design that can reunify the geometric and wave acoustic approaches for simultaneous multifunctionalities, if realized, would contribute significantly to applications that require novel acoustic wave manipulators. Recently, a waveguide material designed based on the conformal transformation method made progress towards such reunification in an optical system, demonstrating concurrent geometric optics and wave optic phenomena [33]. This conformal transformation concept offers a simpler and straightforward approach to those who would like to design unified wave manipulation solution that considers different methodologies. As an important member of the classic wave family, the acoustic wave system can also enjoy the extension of conformal transformation mapping to join the geometric approach with wave acoustics. However, to the best of our knowledge, such topic has not been explored yet. On the other side, conformal transformation acoustics has already been proposed to facilitate the implementation of the acoustic devices made from isotropic materials [34], offering a straightforward yet powerful method to design and implement acoustic metamaterials. More importantly, it has great potential in designing acoustic devices that can take geometrical acoustics and wave acoustics into consideration at the same time. The required constitutive parameters would be much simplified.

In this work, we present a multifunctional acoustic Mikaelian lens designed with conformal transformation acoustics. Mikaelian lens is a gradient medium with a hyperbolic secant refractive index profile that was first devised by Mikaelian and Prokhorov [35]. Recently, many structures have been designed to realize this kind of lens, such as periodic arrays of metal wires [36], square patch arrays [37], and pentamode material [38]. Here, exponentially conformally mapped from Maxwell's fish-eye lens, the Mikaelian lens is realized with a two-dimensional nonhomogeneous cross-channel acoustic metamaterial whose isotropic unit cells are designed to have different side openings to provide flexible phase modulation and nonresonant dispersion. We experimentally demonstrate the capability of such acoustic Mikaelian lens to simultaneously generate sound wave guiding and acoustic Talbot effect, which are normally two distinct ways to demonstrate wave manipulation from the aspects of geometrical and wave acoustics. The Talbot effect is a function of Fresnel diffraction for wave acoustics, which was first discovered and explained in the context of classical diffraction optics [39, 40] and then attracted some attention to be paid in acoustic wave systems [41].

## 2. Results and Discussion

### 2.1. Mikaelian Lens Based on the Conformal Transformation Method

**Figure 1:**
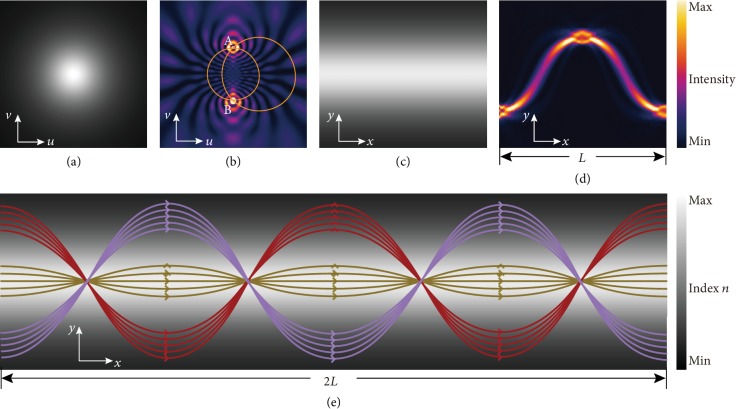
Mapping Maxwell's fish-eye lens to Mikaelian lens. (a) The refractive index distribution of two-dimensional Maxwell's fish-eye lens. (b) Acoustic wave propagation inside the fish-eye lens. Rays emitted from point A will travel along the orange lines and converge at point B. (c) The refractive index distribution of the mapped two-dimensional Mikaelian lens. (d) Acoustic wave propagation inside the Mikaelian lens for a single period. (e) The predicted analytical propagating trajectories for beams with different incident positions (indicated by different colors) inside the Mikaelian lens. In all the plots, (*u*, *v*) indicates the coordinate vector in virtual space and (*x*, *y*) represents that in physical space.

Maxwell's fish-eye lens is a type of imaging device to transform a point source on the boundary of the lens into a focus at the diametrically opposite side of the lens so that the fish-eye lens can be regarded as a perfect imaging instrument. Here, 2D Maxwell's fish-eye lens with a gradient index profile is considered, in which the refractive index varies according to *n* = *α*/(1 + *r*^2^), where *r* is the distance from the center of the lens and *α* is the refractive index at the center [42]. The refractive index profile of the fish-eye lens is given in Figure 1(a), which decreases gradually from the center to the outside. Inside this fish-eye lens, the ray paths are circles emitted from point A and then converged at point B, as shown in Figure 1(b). As the relationship between the refractive index distributions of virtual and physical space being *n*_*z*_ = *n*_*w*_|*dw*/*dz*|, assuming that the index profile *n*_*w*_(*u*, *v*) in virtual space takes a form of *α*/(1 + *u*^2^ + *v*^2^), with an exponential conformal mapping *w*(*u*, *v*) = exp(−*βz*(*x*, *y*)), the corresponding index distribution in physical space would be (see Supplementary Materials for details)

(1) $$\begin{array}{c} n_{z}\left(x,y\right)=n_{0}\mathrm{sech}\left(\beta y\right), \end{array}$$

where *n*_0_ = *αβ*/2 is the maximum refractive index along the central line and *β* is the gradient coefficient which can be utilized to determine the width of the lens. The mapped region of our acoustic Mikaelian lens is ribbon-like with length *L* = 2*π*/*β*, as is shown in Figure 1(c). The color change indicates that the gradual refractive index decreases from the lens center line towards both side ends. Inside the mapped Mikaelian lens, the ray would propagate along a sine-like path, as illustrated in Figure 1(d). For a narrow incident sound beam, the periodically repeated sinusoidal propagation path in our Mikaelian lens can also be predicted, as illustrated in Figure 1(e). The analytical beam trajectory can be derived from the index profile in (1) [43]:

(2) $$\begin{array}{c} y\left(x\right)=\frac{\sinh^{-1}\left(\mu_{0}H\left(x\right)\right)}{\beta }, \end{array}$$

where *H*(*x*) = cos(*βx*), *μ*_0_ = sinh(*βy*_0_), and *y*_0_ is the incident position at *x* = 0. Therefore, once the entry position *y*_0_ is determined, the propagating trajectory of any normal incident beam can be predicted. Such wave guiding effect is a typical geometric acoustic phenomenon. On the other hand, for a grating incident source, all the waves transmitted through different windows would diffract and interfere with each other, which can generate effects in wave acoustics, for example, Talbot effect. The inhomogeneous refractive index distribution in the conformal Mikaelian lens adds more degrees of freedom to manipulate the sound waves, which can take both the ray trajectories and wave phenomena into consideration.

### 2.2. Cross-Channel Acoustic Metamaterial for Mikaelian Lens

**Figure 2:**
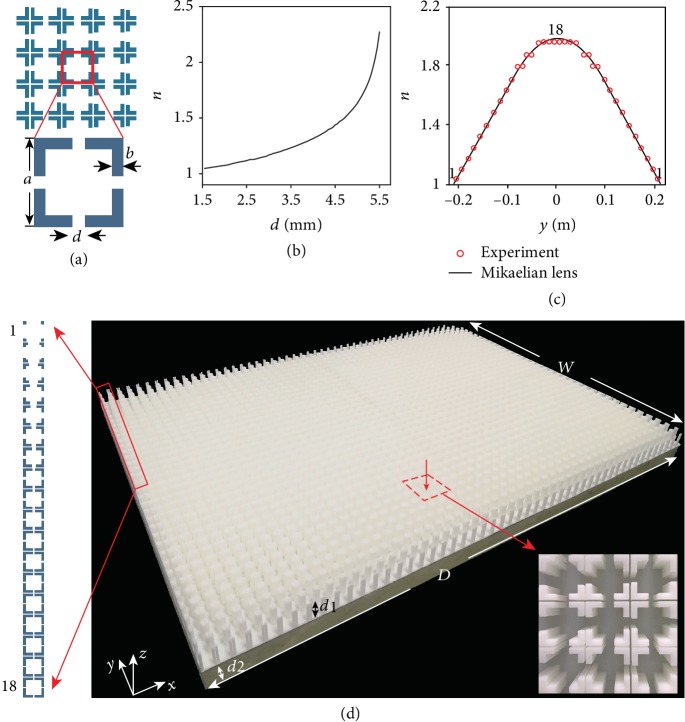
The acoustic metamaterial Mikaelian lens. (a) The two-dimensional cross-channel acoustic metamaterial. For each unit cell, length *a* = 1.14 cm and thickness *b* = 1.5 mm. Slit opening size *d* is used to tune the refractive index. (b) Change of refractive index with the metamaterial unit cell slit opening size *d*. (c) The perfect refractive index profile of Mikaelian lens and discrete indices used for sample fabrication. (d) The fabricated sample. It is comprised of 36 rows and 50 columns. The height of the cells *d*_1_ = 1.8 cm and the thickness of the polymethyl methacrylate plate *d*_2_ = 2 cm. On the left is the top view schematic of the 18 discrete unit cells in the red box; the inset is the detail view of the sample structure.

Our Mikaelian lens is realized with a new cross-channel acoustic metamaterial. As illustrated with a top view 2D model in Figure 2(a), the unit cell of this new acoustic metamaterial is designed from a square shape with side length *a* and side thickness *b*. Slit openings of length *d* are cut at the center of all four side walls. The metamaterial unit cells are center symmetric so that the isotropic requirement of the conformal transformation method can be satisfied. What is important here is that the refractive index of the new acoustic metamaterial can be fine-tuned by simply changing the opening size *d*. In this case, the metamaterial unit cells behave as resonators, whose performance and corresponding effective refractive index are governed by the slit size *d*. Such a capability can be evidently witnessed by the numerical simulation results presented in Figure 2(b), where the value of side length *a* and tube thickness *b* is fixed. Here, the effective refractive indices are extracted based on effective medium theory [44]. The change of the extracted refractive index can be clearly observed when we tune the slit opening size *d*. As anticipated, the refractive index gradually increases with *d*.

A perfect Mikaelian lens calls on a continuous index profile predicted by (1) as shown in Figure 2(c). However, such scenario is extremely hard to realize in practice since the metamaterial unit cell size cannot be infinitely small. Therefore, we discretize the continuous refractive index distribution and construct a two-dimensional acoustic metamaterial with 36 × 50 unit cells, as shown in Figure 2(d). It is located inside a 2 cm high, 60 cm long, and 43.2 cm wide waveguide to form the complete Mikaelian lens. The operating frequency of the lens is set at 3 kHz. The center distance between the adjacent metamaterial unit cells is 12 mm. The side wall length *a* and the wall thickness *b* of all metamaterial unit cells are, respectively, 11.4 mm and 1.5 mm, much smaller than the sound wavelength in air. The heights of all metamaterial unit cells are 1.8 cm, to guarantee the pressure invariant along the *z* axis. As shown in Figure 2(d), the unit cells have uniform structures along the *x* axis. But along the *y* axis, each unit cell has their own slit openings value *d* so that the effective index distribution would satisfy the Mikaelian lens requirement calculated based on (1). With the *n*_0_ and *β* in (1) chosen to be 2.03 and 6 based on the maximal refractive index obtained with the acoustic metamaterial, a corresponding refractive index change from 1.03 at both ends to 2.03 in the middle at *y* = 0 can be obtained. In our setup, the effective index distribution of Mikaelian lens is equally discretized into 18 parts away from *y* = 0. However, to satisfy the resolution accuracy requirement of 3D printing, the space between the adjacent structures cannot be smaller than 0.1 mm. Therefore, we choose to assign 13 different indices to the 18 discrete metamaterial unit cells, as sketched in Figure 2(d). Please refer to [Supplementary-material supplementary-material-1] in Supplementary Materials for detail.

### 2.3. Sound Guiding Effect

**Figure 3:**
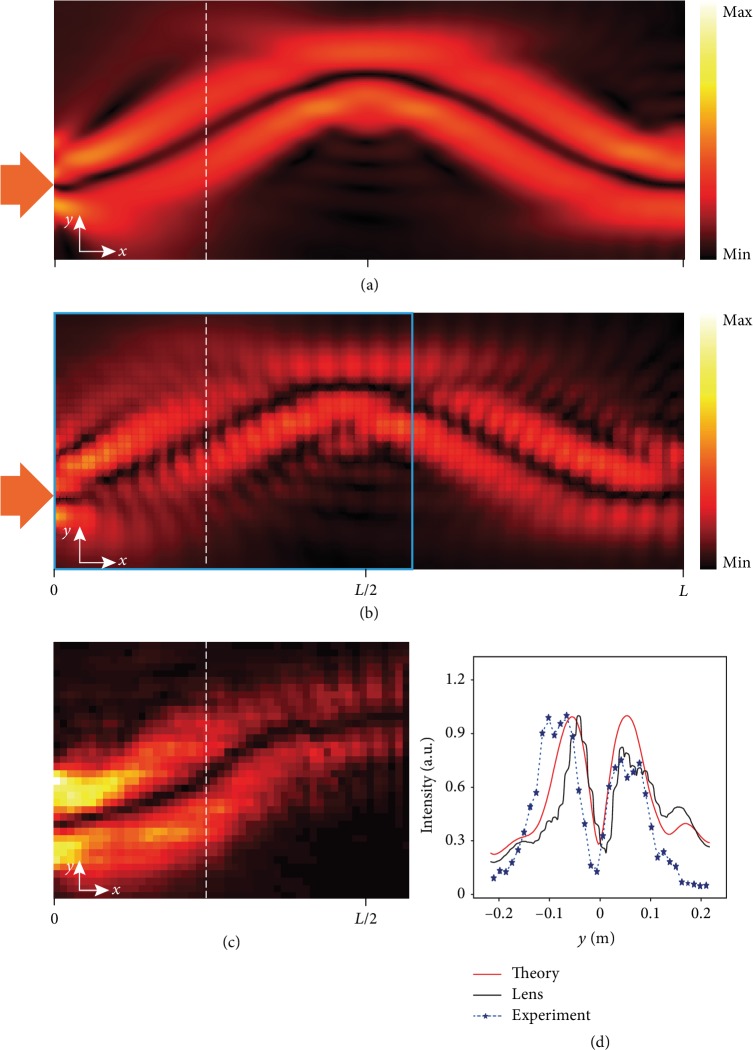
Sound guiding effect. (a) The simulated absolute sound pressure distribution of Airy beam incident on the Mikaelian lens with the perfect continuous refractive index distribution. (b) The simulated acoustic field distribution of Airy beam incident on the designed Mikaelian lens with the real cross-channel metamaterial structure. (c) The experimentally measured absolute sound pressure distribution for the area marked with the cyan box in (b). (d) The normalized |*p*| profiles for the dotted lines marked in (a–c).

The wave guiding effect of confining sound propagation along a specific path is an attractive phenomenon in geometric acoustics, by which the guided trajectory of an incident beam can be analytically predicted [43]. It enables the sound beams to propagate along a tailored fashion and has a number of potential applications, such as acoustic sensing, imaging, and particle manipulation [45–47]. To demonstrate the wave guiding effect, we use the Airy beam Airy(25*y*) as input. The airy function incident beam can be generated by arranging the phase distribution about its function [45] (please refer to [Supplementary-material supplementary-material-1] in Supplementary Materials). We realize the beam Airy(25*y*) with two planar sources at input side *x* = 0. The phase *φ* for position −0.094 m < *y* < 0 approximates to 0, and *φ* for position ‐0.16 m < *y*<−0.094 m is *π*. In a normal waveguide filled with air, Airy beam propagates along its nondiffractive trajectory with intensity decreasing gradually. However, as shown in Figure 3, when the same Airy beam enters a perfect Mikaelian lens, it will instead travel along a sinusoidal-like path and repeat periodically with a period of *L* = 2*π*/*β*, about 1.047 m. We also conduct full wave simulation with our acoustic metamaterial Mikaelian lens. As presented in Figure 3(b), although the actual structure of acoustic metamaterial unit cells causes some discretization of beam shape, the overall Airy beam trajectory is consistent with the acoustic field distribution shown in Figure 3(a) when we consider the refractive index to be continuously distributed. In experiments, we measured the sound field distribution alongside the lens. The results demonstrated in Figure 3(c) agree well with both simulation outputs, evidently verifying the wave guiding capability of the acoustic metamaterial Mikaelian lens. The normalized absolute pressure profiles at *x* = *L*/2, acquired from the simulations and experiments, are further compared in Figure 3(d). Similar variation pattern and shape can also be clearly observed for three different cases. The position difference of curve peaks is due to our discretization of the continuous Mikaelian lens index profile and the inevitable intrinsic loss in experiment. It is worth mentioning that other interesting phenomena within the geometric acoustic scope can also be realized in this acoustic metamaterial waveguide. For example, periodical self-focusing effect with a *L*/2 foci interval can be obtained for a wide plane wave incidence (see Supplementary Materials for the detail).

### 2.4. Talbot Effect

The Talbot effect is a near-field diffraction effect, which is first observed in 1836 [39]. It exhibits repeated field distributions at regular distances away from the structure after a plane wave incidence (referred to as self-imaging). Recently, it has been explored in matter waves [48, 49], plasmons [50], acoustics [41, 51], and optics [33, 52], with potential applications in imaging, wave manipulation, and data transmission [53–55]. Talbot effect of the acoustic wave could benefit the sound transmission alteration and the visualization of surface acoustic wave propagation [51].

**Figure 4:**
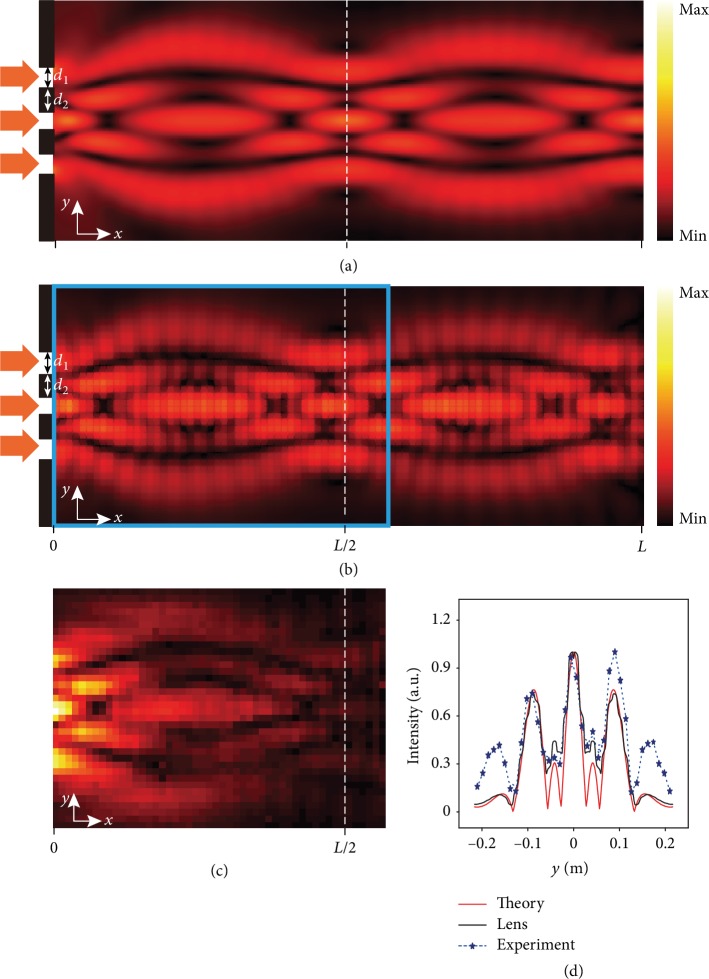
Acoustic Talbot effect. (a) The simulated absolute sound pressure distribution for the lens with the perfect continuous index profile. (b) The simulated acoustic field distribution in the designed Mikaelian lens (*d*_1_ = 4 cm, *d*_2_ = 5 cm, *d*_1_ is the width of each incident beam, and *d*_2_ is the width of the hard boundary between each two incident sources). (c) The experimentally measured absolute sound pressure distribution for the area marked with the cyan box denoted in (b). (d) The normalized |*p*| for the dotted lines marked in (a–c).

For Talbot effect, sending an incident sound beam to our acoustic Mikaelian lens should result in the observation of periodical self-imaging of the incident wave pattern inside the waveguide lens. In our demonstration, we use a three-beam incidence as shown by the red arrows in Figures 4. The width of each incident beam is 4 cm. The interval between each two beams is 5 cm. In a normal waveguide, the grating pattern of the incident source cannot be transferred to far field due to the diffraction loss. However, in our conformal Mikaelian lens, the pattern of input beam can be transferred and recovered at remote locations along the lens. Such capability can be witnessed by the simulation and experimental results presented in Figure 4. In simulation, we consider both continue index profile and discrete index profile realized with a real cross-channel acoustic metamaterial structure. For both cases, the self-imaging of the three narrow incident beams can be observed, at around *x* = *L*/2. This Talbot effect can also be observed periodically at *x* = *nL*/2, where *n* is a positive integer. The experimentally measured absolute sound pressure distribution inside the metamaterial Mikaelian lens is shown in Figure 4(c). The whole process of replicating the incident source pattern can be clearly observed. The reimaging position also matches well with the simulation result. We further plot the normalized absolute sound pressure variations along the first self-imaging plane *x* = *L*/2 in Figure 4(d). The experimental result matches well with the simulation outputs. The slight difference of amplitudes is due to the imperfect absorption boundary and the fabrication error.

### 2.5. Sound Guiding Effect and Talbot Effect over Spectrum

**Figure 5:**
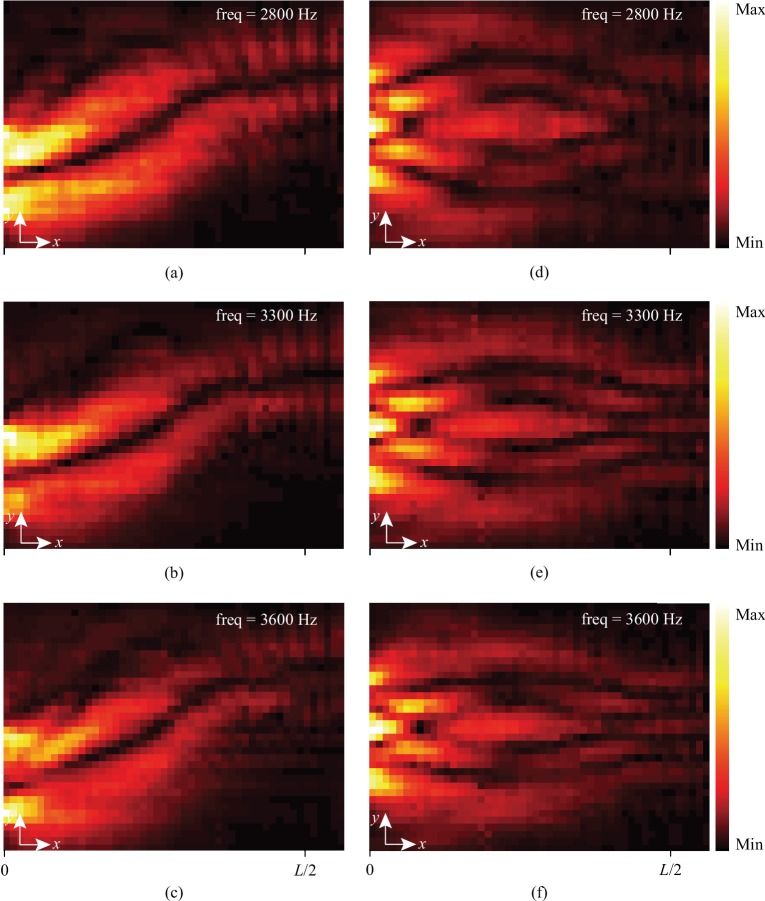
Sound guiding and acoustic Talbot effects at different frequencies. (a–c) The experimentally measured sound guiding effect for the operating frequencies of 2800 Hz, 3300 Hz, and 3600 Hz, respectively. (d–f) The experimentally measured Talbot effect for the operating frequencies of 2800 Hz, 3300 Hz, and 3600 Hz, respectively.

Our acoustic Mikaelian lens acquires desired effective refractive indices through adjusting the slit opening size of the cross-channel metamaterial. Such capability can be extended over the spectrum when the operating frequency is designed to be well away from the resonant frequency of the metamaterial unit cell. Within the targeted frequency range (2800 Hz-3600 Hz), the effective refractive index is almost nondispersive, guaranteeing the broadband performance of our proposed lens (see Supplementary Materials for details). Therefore, compared with the similar case in optics, the proposed cross-channel acoustic metamaterial can provide better spectral performance in wave manipulation for acoustic applications. In experiments, we tested the geometric acoustics and wave acoustic functionalities of our acoustic Mikaelian lens at multiple different frequencies. The relatively broadband wave guiding and Talbot effect demonstrated in Figure 5 are consistent with each other and show great agreement with the simulation results in Supplementary Materials. The sound guiding effect and Talbot self-imaging can be clearly observed even when the operating frequency increases from 2800 Hz to 3600 Hz.

## 3. Discussion

Based on conformal transformation acoustics, we have designed and demonstrated an isotropic acoustic Mikaelian lens constructed with cross-channel metamaterial. Our experimental results clearly show that this lens is capable of manipulating acoustic wave concurrently with respect to both geometrical acoustics and wave acoustics. It is worth mentioning that, although resonances contribute to the acoustic metamaterial index modulation, the operating frequency is much lower than the fundamental resonant frequency. Therefore, the proposed acoustic Mikaelian lens can enable the Airy beam sound guiding and Talbot effect within a relatively wide frequency range without drastic dispersion, which makes it a better platform compared with the case in optics [33].

The conformal transformation method adopted in our work offers a powerful tool to explore more fascinating sound phenomena. For example, the whispering gallery modes with high Q factor and directional emission may help to attain directional coherent acoustic beam. The methodology described in our work can be extended to a wide range of wave-physics domains, opening new potential prospects for fundamental studies and practical applications.

## 4. Materials and Methods

All simulations were performed using COMSOL Multiphysics. The materials used to fabricate the waveguide (polymethyl methacrylate) and all acoustic metamaterial unit cells (photopolymer), due to the huge impedance mismatch between the material (*ρ* = 1190 kg/m^3^, *c* = 1640 m/s) and air, are considered rigid media in the full wave simulations. The outer boundary condition is plane wave radiation to simulate the experimental condition. The Mikaelian lens consisting of an array (36 × 50) of metamaterial unit cells is fabricated with photopolymer by 3D printing with a manufacturing precision of 0.1 mm. The size of each unit cell is well tailored to be 1.2 cm to leave enough space in the center for the experimental measurement. The height of the unit cells is 1.8 cm, and a base plate is printed to hold all the unit cells together.

The experimental measurements were conducted in a waveguide, with metamaterial Mikaelian lens sample sandwiched between two polymethyl methacrylate plates. The overall length and width of the waveguide is 60 cm and 43.2 cm, respectively. Sound-absorbing cotton was placed around the sample. The incident sound waves were generated by a loudspeaker array driven by a function generator. The sound fields inside the Mikaelian lens were acquired by moving the B&K 4138-A-015 microphone to collect the signal with resolution of 1.2 cm.

## Supplementary Materials

Supplementary Materials Section S1: conformal transformation method. Table S1: the slit sizes for all the units to generate the discretized refractive index of the Mikaelian lens. Figure S1: the acoustic field distributions for different incident sources. Figure S2: self-focusing effect. Figure S3: the refractive index profiles for different slit sizes and frequencies. Figure S4: wave guiding and Talbot effect for different frequencies.
