# Supplementary material for: Conformally Mapped Multifunctional Acoustic Metamaterial Lens for Spectral Sound Guiding and Talbot Effect
Source: Research (Wash D C). 2019 Nov 12;2019:1748537. doi: 10.34133/2019/1748537 (PMC6944515; doi:10.34133/2019/1748537)
Supplement: Supplementary Materials — Section S1: conformal transformation method. Table S1: the slit sizes for all the units to generate the discretized refractive index of the Mikaelian lens. Figure S1: the acoustic field distributions for different incident sources. Figure S2: self-focusing effect. Figure S3: the refractive index profiles for different slit sizes and frequencies. Figure S4: wave guiding and Talbot effect for different frequencies. [file 1748537.f1.docx]

**Supplementary Materials**

**Conformally Mapped Multifunctional Acoustic Metamaterial Lens for Spectral Sound Guiding and Talbot Effect**

**He Gao^1^, Xinsheng Fang^2^, Zhongming Gu^1^, Tuo Liu^1,3^, Shanjun Liang^1,3^, Yong Li^2 *^,**

**Jie Zhu^1,3 *^**

^1^*Department of Mechanical Engineering, The Hong Kong Polytechnic University, Hung Hom, Kowloon, Hong Kong SAR, China.*

^2^*Institute of Acoustics, School of Physics Science and Engineering, Tongji University, Shanghai 200092, PR China.*

^3^*The Hong Kong Polytechnic University Shenzhen Research Institute, Shenzhen, 518057, PR China.*

^*^To whom correspondence should be addressed. Emails: [jiezhu@polyu.edu.hk](mailto:jiezhu@polyu.edu.hk) (J. Z.); yongli@tongji.edu.cn (Y. L.).

**1. Conformal transformation method**

Conformal mapping locally keeps the angle of two random lines unchanged during the mapping between the virtual space and physical space [1]. Suppose the complex number $w=v+iu$ indicates the virtual space, while the physical space is denoted by $z=y+ix$. For a waveguide with a gradient index distribution $n_{z}(x,y)$, the Helmholtz equation is:

$(\partial_{x}^{2}+\partial_{y}^{2}+n_{z}^{2}k^{2})\Phi=(4\partial_{z}^{*}\partial_{z}+n_{z}^{2}k^{2})\Phi=0$ (S1)

where *k* is the wave number. By employing the conformal mapping $w=w(z)$, the Helmholtz equation for the virtual space would be changed to:

$(4\partial_{w}^{*}\partial_{w}+n_{w}^{2}k^{2})\Phi=0$ (S2)

Since this conformal mapping satisfies Cauchy-Riemann condition, with (S1) and (S2), the relationship between refractive index distributions of virtual and physical space can be obtained:

$n_{z}=n_{w}\left|\frac{dw}{dz}\right|$ (S3)

Once the refractive index profile in virtual space $n_{w}(u,v)$ and the relationship between these two spaces are given, the corresponding refractive index distribution in physical space would be achieved [1]. In this work, the mapping relationship between the virtual and physical space is chosen to be $w=\exp(-\beta z)$, so that the coordinate relationship would be $u=\text{-}\exp(-\beta y)\sin(\beta x)$ and $v=\exp(-\beta y)\cos(\beta x)$. As the refractive index distribution in the virtual space $n_{w}=\frac{\alpha}{1+u^{2}+v^{2}}$, with (S3), we can derive the mapped index profile in physical space $n_{z}(x,y)=n_{0}\mathrm{sech}(\beta y)$.

**2. Table S1. The slit sizes for all the units to generate the discretized refractive index of the Mikaelian lens**


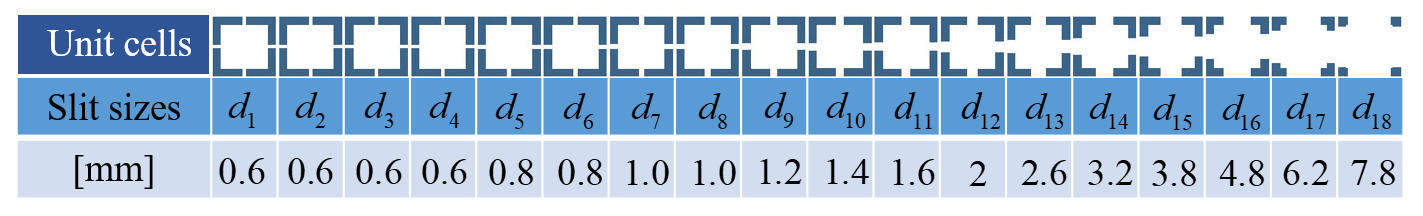


1. **The acoustic field distributions for different incident waves**

In a waveguide which has air as the background medium, the incident Airy beam would propagate along its bending trajectory and the intensity would decrease gradually, as shown in Figure S1(a), in which the inset is the **according** Airy function plot. Meanwhile, when a grating source acts as the incident wave, the incident field pattern cannot be maintained and transferred to the far field, as is depicted in Figure S1(b).


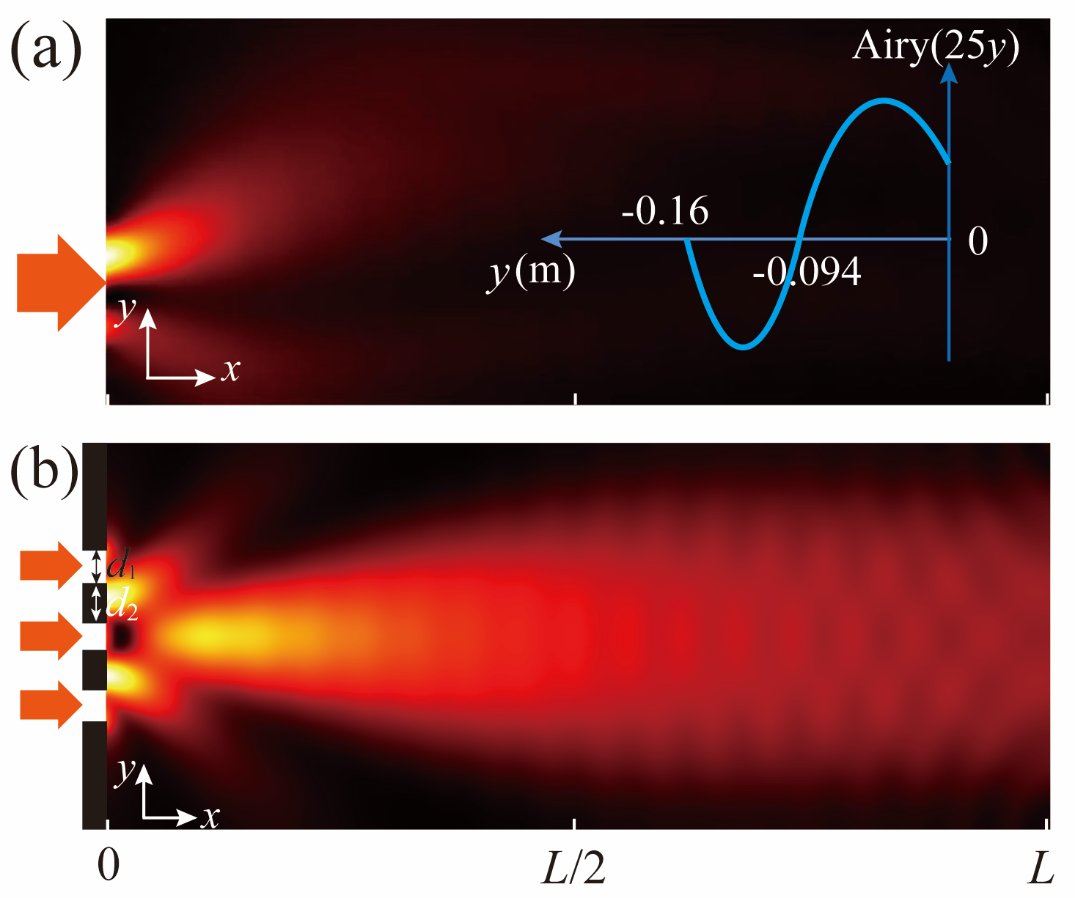


Figure S1. The acoustic field distributions for different incident sources. (a) Airy beam incident on the waveguide filled with air (the inset is the according Airy function). (b) A periodic wave incident on the same waveguide filled with air.

1. **Acoustic self-focusing effect**

Sound guiding is one of the fantastic phenomena within the geometric acoustics scope. Besides, by redesigning the incident sources, more interesting phenomena in this field can be observed in the same waveguide, for example, the self-focusing effect. To obtain this effect, a plane wave is required to act as the incident source, which can be generated by a loudspeaker array. Once this plane wave incident on the Mikaelian lens, the sound energy would be automatically focused at the positions $x=(2n-1)L/4$. *n* is a random positive integer and $L\text{=}1.074\text{ m}$ being the period of Mikaelian lens. The theoretical acoustic field distribution in this lens is given in Figure S2(a). In addition, the simulation of the acoustic field has been conducted in our designed waveguide, as depicted in Figure S2(b), which shows the similar self-focusing property with Figure S2(a). By comparing Figures S2(a) and S2(b), a slight difference of the focal positions can be observed, which is caused by the discretization of the refractive index profile. Furthermore, this self-focusing property has been verified experimentally. The measured result of the same region in Figure S2(b) (encircled by the cyan box) is given in Figure S2(c). To have a better view of this self-focusing effect, the normalized $\left|p\right|$ profiles along the central lines of the first foci ($x=L/4$) in Figures S2(a-c) are depicted in Figure S2(d) respectively. The focal positions along $y$ direction have a good agreement and their energy distributions possess the similar tendency, which confirms that the self-focusing effect can be achieved by our proposed design.


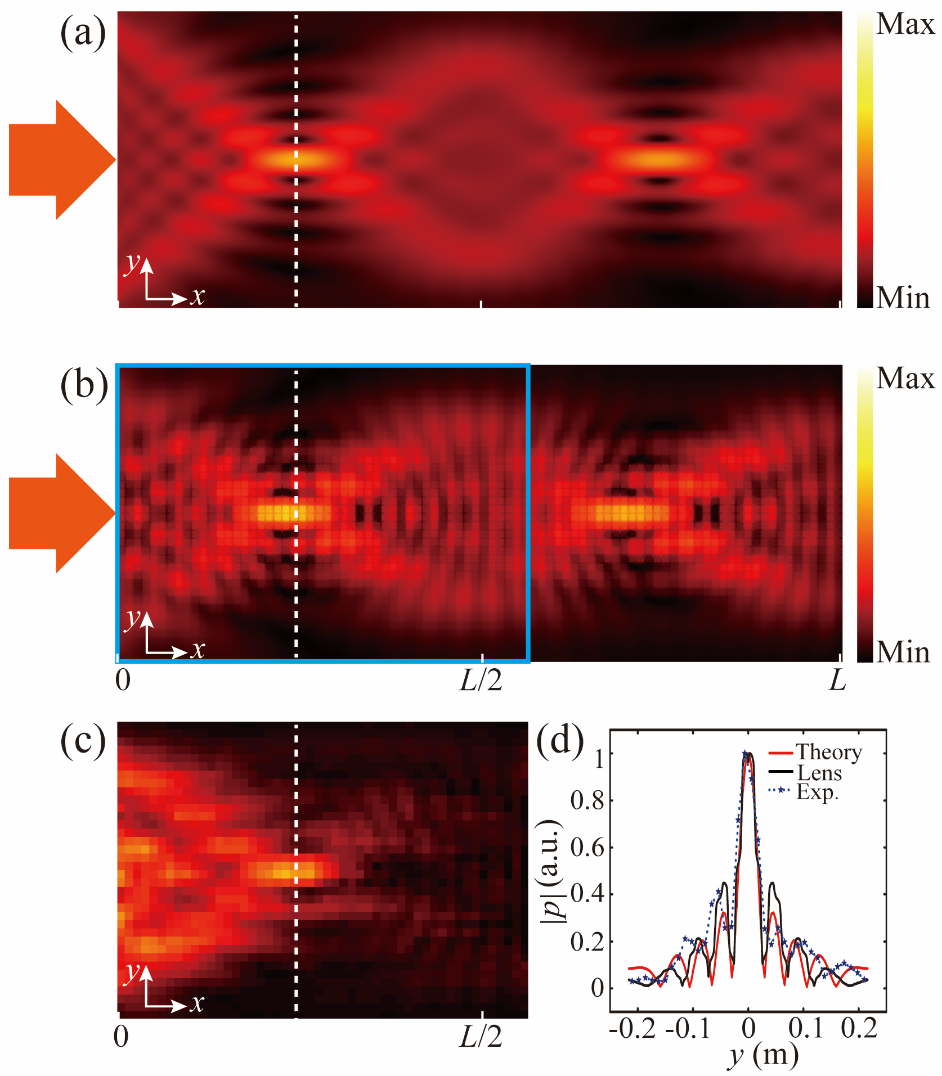


Figure S2 Self-focusing effect. (a) The acoustic field distribution with the theoretically continue index profile. (b) The acoustic field distribution in the designed Mikaelian lens. (c) The measured acoustic field distribution in the cyan box illustrated in Figure S2(b). (d) The normalized $\left|p\right|$ profiles for the theory, designed lens and experiment at the position denoted by the white dotted lines in Figures S2(a-c) respectively.

1. **Broadband property of the designed structure**

The effective refractive indices can be controlled by adjusting the opening size of the slit metamaterial within a relatively broad frequency range well away from the resonant frequencies. To confirm this, the retrieved effective properties for unit cells with three different slit sizes ($d=0.6 mm, 2 mm,7.8 mm$) used in our design are presented, as shown in Figure S3(a). For the structures with large slits ($d=7.8 mm, 2 mm$), the refractive indices almost keep a constant as the frequency increases from 2 kHz to 4 kHz, the one with a smaller slit ($d=0.6 mm$) has a slight dispersion in the high frequency region, which is caused by the decreased resonant frequency but has little impact on the validness of our scheme. Furthermore, the refractive index profiles of all the units at different frequencies are depicted in Figure S3(b). As the frequency increases from 2800 Hz to 3600 Hz, the refractive indices almost remain unchanged and only slight differences are observed around the position $y=0$.


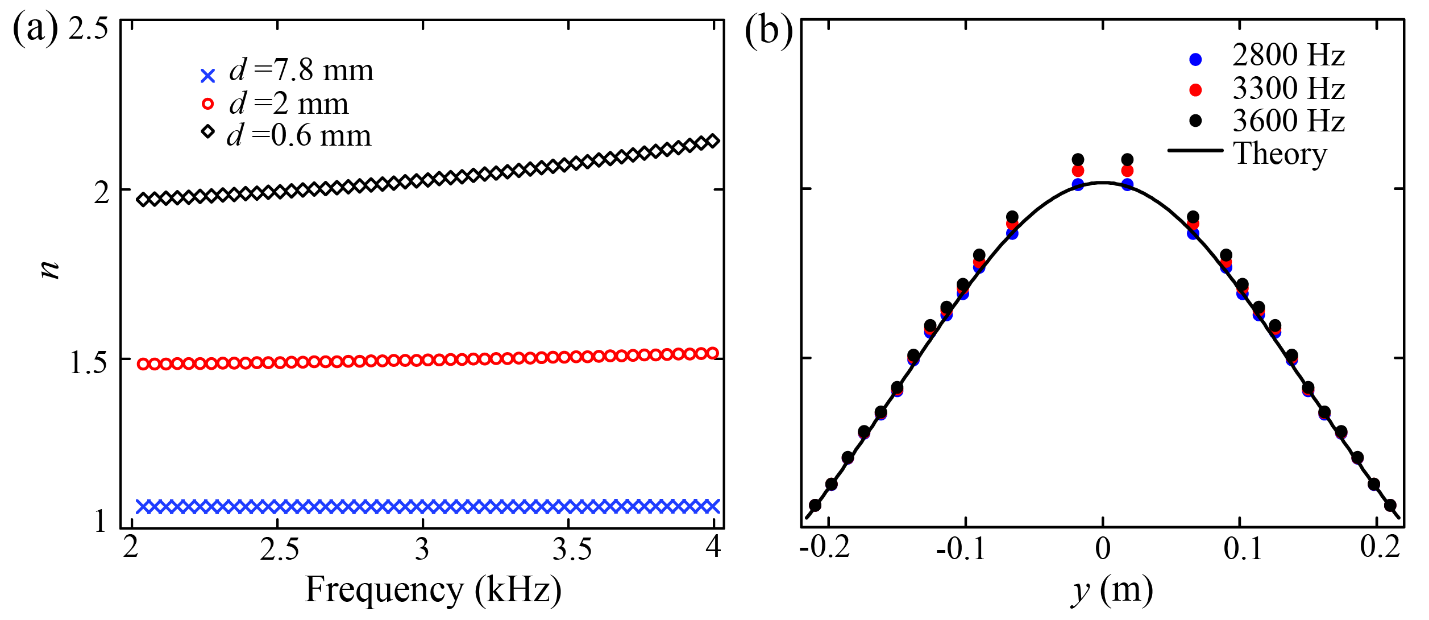


Figure S3 The refractive index profiles for different slit sizes and frequencies. (a) The effective refractive indices as a function of frequency for different slit sizes. The opening sizes are 7.8 mm (blue crosses), 2 mm (red circles) and 0.6 mm (black squares) respectively. (b) The refractive index profiles for different frequencies. The black solid line represents the theoretically continue index distribution of Mikaelian lens, the blue, red and black circles indicate the index distributions for frequency being 2800 Hz, 3300 Hz and 3600 Hz respectively.

1. **Wave guiding effect and Talbot effect for different frequencies**

As the refractive indices of designed slit metamaterials almost remain a constant within a certain frequency range, here we have demonstrated both the wave guiding and Talbot effect for frequencies being 2800 Hz, 3300 Hz and 3600 Hz respectively. Figures S4(a-c) show the simulated acoustic fields for the wave guiding effect in the three cases. Similarly, the results for the Talbot effect are given in Figures S4(d-f). We can see both the wave guiding and Talbot effect can always be clearly observed as the frequency increases from 2800 Hz to 3600 Hz which demonstrate the relatively broadband property of our designed Mikaelian lens.


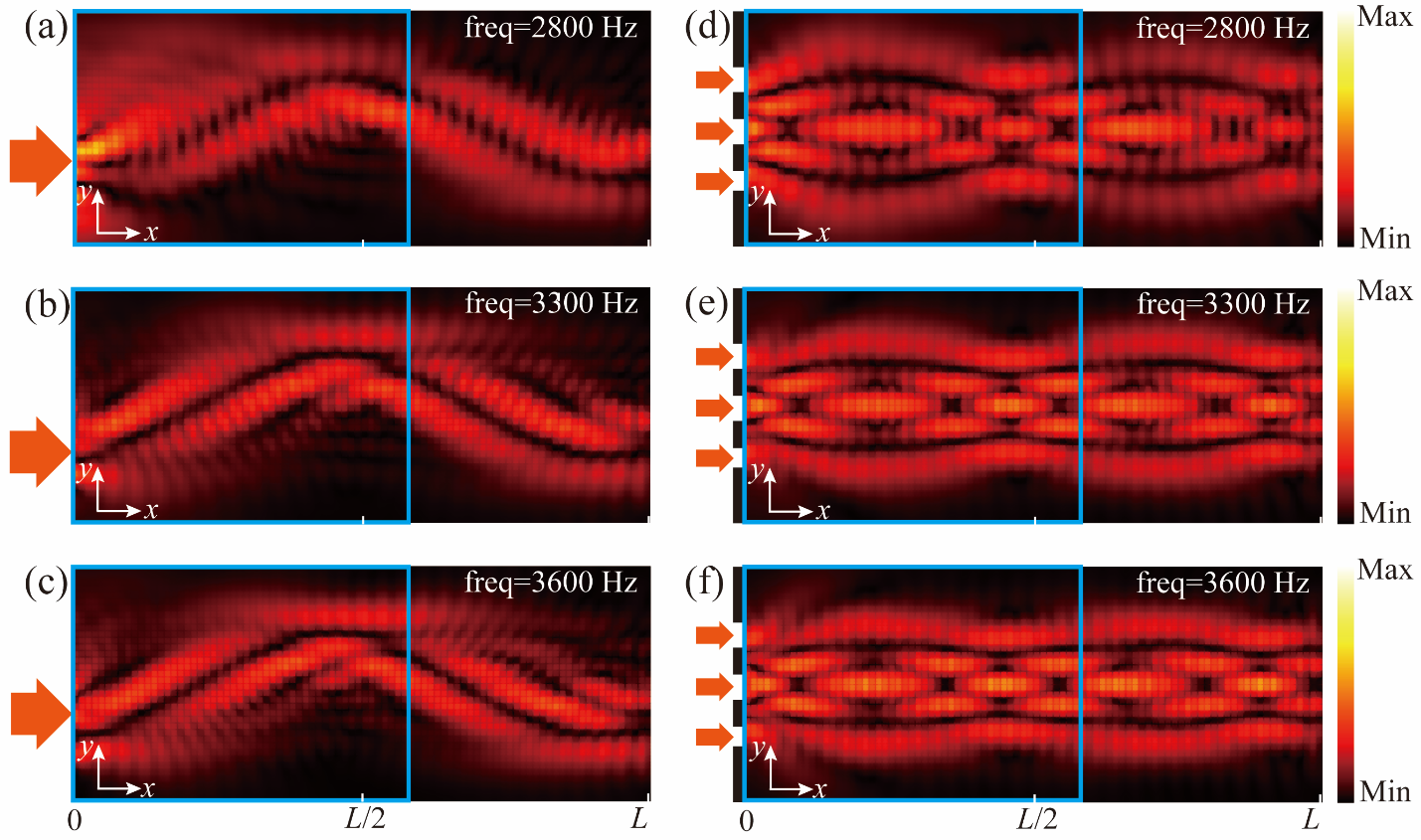


Figure S4 Wave guiding and Talbot effect for different frequencies. (a-c) The simulated acoustic field distributions for the wave guiding effect at frequencies being 2800 Hz, 3300 Hz and 3600 Hz respectively. (d-f) The simulated acoustic field distributions for the Talbot effect at frequencies being 2800 Hz, 3300 Hz and 3600 Hz respectively.

# References

1. U. Leonhardt, "Optical conformal mapping," *Science*, vol. 312, no. 5781, pp. 1777-1780, 2006.
